# Cell-Penetrating Recombinant Peptides for Potential Use in Agricultural Pest Control Applications

**DOI:** 10.3390/ph5101054

**Published:** 2012-09-28

**Authors:** Stephen R. Hughes, Patrick F. Dowd, Eric T. Johnson

**Affiliations:** 1 Renewable Product Technology Research Unit, National Center for Agricultural Utilization Research (NCAUR), Agricultural Research Service (ARS), United States Department of Agriculture (USDA), 1815 North University Street, Peoria, IL 61604, USA; 2 Crop Bioprotection Research Unit, National Center for Agricultural Utilization Research (NCAUR), Agricultural Research Service (ARS), United States Department of Agriculture (USDA),1815 North University Street, Peoria, IL 61604, USA; Email: patrick.dowd@ars.usda.gov (P.F.D.); eric.t.johnson@ars.usda.gov (E.T.J.)

**Keywords:** biological pest control, cell-penetrating peptides, spider venom, recombinant lycotoxin

## Abstract

Several important areas of interest intersect in a class of peptides characterized by their highly cationic and partly hydrophobic structure. These molecules have been called cell-penetrating peptides (CPPs) because they possess the ability to translocate across cell membranes. This ability makes these peptides attractive candidates for delivery of therapeutic compounds, especially to the interior of cells. Compounds with characteristics similar to CPPs and that, in addition, have antimicrobial properties are being investigated as antibiotics with a reduced risk of causing resistance. These CPP-like membrane-acting antimicrobial peptides (MAMPs) are α-helical amphipathic peptides that interact with and perturb cell membranes to produce their antimicrobial effects. One source of MAMPs is spider venom. Because these compounds are toxic to insects, they also show promise for development as biological agents for control of insecticide-resistant agricultural pests. Spider venom is a potential source of novel insect-specific peptide toxins. One example is the small amphipathic α-helical peptide lycotoxin-1 (Lyt-1 or LCTX) from the wolf spider (*Lycosa carolinensis*). One side of the α-helix has mostly hydrophilic and the other mainly hydrophobic amino acid residues. The positive charge of the hydrophilic side interacts with negatively charged prokaryotic membranes and the hydrophobic side associates with the membrane lipid bilayer to permeabilize it. Because the surface of the exoskeleton, or cuticle, of an insect is highly hydrophobic, to repel water and dirt, it would be expected that amphipathic compounds could permeabilize it. Mutagenized lycotoxin 1 peptides were produced and expressed in yeast cultures that were fed to fall armyworm (*Spodoptera frugiperda*) larvae to identify the most lethal mutants. Transgenic expression of spider venom toxins such as lycotoxin-1 in plants could provide durable insect resistance.

## 1. Introduction

The cell membrane is the barrier that protects living cells from the surrounding environment, generally allowing the movement of only small molecules into the cell. The hydrophobic nature of cell membranes makes them impenetrable to most peptides, proteins, and oligonucleotides. However, it was eventually observed that certain peptides had the ability to pass through cell membranes. These molecules have been called cell-penetrating peptides (CPPs). In general, CPPs are short (less than 35 amino acid residues), water-soluble and partly hydrophobic, and/or polybasic peptides with a net positive charge at physiological pH. Their ability to translocate across cell membranes is attributed to their highly cationic and partly hydrophobic structure. This class of peptides has been extensively studied for several decades, but the mechanism(s) by which CPPs enter the cells is not completely understood [1]. Another class of peptides that have similar characteristics to CPPs and that in addition possess antimicrobial properties is being investigated for use as antibiotics that have a reduced risk of causing resistance [2]. These CPP-like membrane-acting antimicrobial peptides (MAMPs) are produced by many organisms as a defense against microbial pathogens. They share several features with CPPs. They are short and have a high positive charge. In addition, most have amphipathic α-helical structures that interact with and perturb cell membranes to produce their antimicrobial effects [3,4]. They target a wide range of bacteria and fungi and these pathogens do not seem to readily develop resistance [5]. Because several of these compounds are also toxic to insects, they also show promise for development as biopesticides for control of insecticide-resistant agricultural pests.

Spider venom is injected into insect prey to subdue it, and is a potential source of novel MAMP insect-specific peptide toxins [6]. An example is the small amphipathic peptide lycotoxin-1 (Lyt-1 or LyTX) from the wolf spider (*Lycosa carolinensis*). Lycotoxin‑1 appears to function as a pore former to increase membrane permeability and to bring about lysis of cells by reducing ion and voltage gradients across membranes [7,8]. The amphipathic α-helical character of the secondary structure of lycotoxin-1 results from lysine repeats that occur every fourth or fifth position in the peptide sequence. Several other pore-forming peptides, including magainins, dermaseptins, and adenoregulin, have a similar lysine motif with characteristic amphipathic α-helical secondary structures. The predicted secondary structure of lycotoxin-1 demonstrates that the majority of the hydrophobic and hydrophilic amino acid residues occur on opposite sides of the helix. The net positive charge of the hydrophilic side of the helix promotes interaction with negatively charged prokaryotic membranes (normal mammalian cell membranes have more positive-charge character) and the hydrophobic side of the helix associates with the membrane lipid bilayer to permeabilize the membrane. A similar mechanism of action might be postulated for its insecticidal properties. The exoskeleton, or cuticle, of an insect, is composed of a protein, polyphenol, water and lipid matrix in which are imbedded chitin nanofibers. The surface of the cuticle is highly hydrophobic, to repel water and dirt [9]. It would be expected that amphipathic compounds of appropriate structure could permeabilize the cuticle of the insects and thus penetrate both hydrophobic and hydrophilic layers, and subsequently move into the aqueous hemolymph and continue to induce cell lysis; thus not requiring injection. Modification of a regulatory peptide to better penetrate the cuticle has resulted in increased efficacy for inhibiting pheromone biosynthesis [10].

The potential use of lycotoxin-1 as a bioinsecticide was investigated by producing recombinant mutant lycotoxin-1 peptides via an amino acid scanning mutagenesis strategy and screened in high throughput for ability to kill fall armyworms, a significant cause of damage to corn and other crops in the United States. Yeast cultures expressing the recombinant peptide toxins were fed to fall armyworm (*Spodoptera frugiperda*) larvae to identify the mutant toxins with greatest lethality. The most lethal mutations appeared to increase the ability of the toxin α-helix to interact with insect cell membranes and disrupt the membranes by forming pores [11]. 

## 2. Diverse Applications of CPPs and CPP-Like Molecules

Many drugs are large hydrophilic molecules showing major limitations for their penetration through the cell membrane. A group of short peptides designated CPPs have been discovered that may serve as delivery vectors for large molecules, for example, therapeutic compounds [1,12]. The main feature of CPPs is that they are able to penetrate the cell membrane at low micromolar concentrations *in vivo* and *in vitro* without using chiral receptors [1,13]. The first members of this class of peptides were the human immunodeficiency virus type 1 (HIV-1) encoded Tat peptide described in 1988 and the amphiphilic *Drosophila antennapedia* homeodomain-derived penetratin peptide (pAntp) discovered three years later. They are the most extensively studied of all CPPs [1]. It was demonstrated that CPP Tat fused to enhanced Green Fluorescent Protein facilitates its internalization and transepithelial transport into the columnar cells from intact larval midgut tissue of *Bombyx miori* (Lepidoptera: Bombycidae). These results offer the possibility of effective oral delivery of bioinsecticidal molecules to targets located both within the insect gut epithelium and behind the gut barrier, in the hemocoel compartment, provided cargo molecules selectively acting on the pest species to be controlled are available [14]. During the ensuing decades, a vast number of CPPs and other similar cytolytic and membrane-acting peptides have been discovered and their mechanisms of action examined [1,15]. The correlation between sequence and function is still not entirely clear. A review of the mechanisms of six cell-penetrating, antimicrobial, and cytolytic peptides found that although their specificity varied, all of these peptides formed α-helices when bound to membranes and they all showed remarkable specificity for the target membrane or organism. The authors concluded that the sequence specifies the mechanism only indirectly through the thermodynamics of peptide insertion into the bilayer medium from the surface-bound state. This would provide an explanation for the specificity of antimicrobial peptides because cationic peptides would bind better to the anionic membranes of most bacteria than to the neutral membranes of eukaryotic cells [15].

The development of drug-resistant microbes is an increasingly serious public health problem worldwide. Recently widespread antibiotic resistance has emerged in clinically important bacterial pathogens such as *Staphylococcus aureus*, *Streptococcus pneumoniae*, and *Enterococcus faecalis*. Attention has turned to the CPP-like MAMPs as compounds that can be used against drug-resistant microbes. MAMPs are active against a wide range of Gram-negative and Gram-positive bacteria, including drug-resistant strains. Their mechanism of interaction with cell membranes does not involve specific protein binding sites, which reduces the likelihood of inducing drug resistance. Studies are underway to understand the structure-function relationship of these peptides and develop them into useful antibiotic drugs. These studies have suggested that both pore-forming and non-pore models can be used to describe the function of MAMPs [5]. It is believed that they act by disrupting the integrity of the cell membrane, which causes either a massive membrane failure or smaller-scale defects that dissipate voltage gradients, both resulting in cell death. Much of the evidence for the mechanism of action is based on results using synthetic vesicles. There is a lack of information about the interaction of MAMPs with bacterial membranes. Experiments are needed to compare the effects of these peptides on bacterial and mammalian cells. With this information it will be possible to accelerate development of these CPP-like MAMPs into clinically useful antibiotic drugs [5,6].

## 3. Interest in CPPs and CPP-Like Molecules for Bioinsecticides

It is estimated that insect pests destroy approximately 14% of the World’s crop production [16]. In many cases, natural enemies are not sufficient to control pests adequately [17]. The *Bacillus thuringiensis* (Bt) crystal proteins are examples of insecticidal proteins that have been developed commercially. They are highly effective against a targeted range of species when used as topical pesticides [18,19] and more recently when expressed in transgenic plants to confer inherent pest resistance [20,21,22] for complete control of some insect species, such as European corn borers [23]. Widespread use of host-plant resistance is limited by the availability of cultivars with high levels of resistance to key pest species. With the application of recombinant DNA technology to genetically engineer insect-resistant crop plants, this constraint can be eliminated [20]. Candidate genes are available for insecticidal proteins occurring in nature that are effective against agriculturally important pests but are harmless to non-target species, and their use in transgenic plants has significant potential to provide sustainable crop protection systems [19,20,21].

Spiders are among the most successful terrestrial predators. One reason for their success is the production of highly toxic venom from their venom glands that they employ to subdue prey and deter predators [24]. Of the 800 peptides described in ArachnoServer 2.0 [25], a curated database containing available information on spider-venom peptides and proteins, 136 are insecticidal, with 38 being insect-selective, 34 non-selective and 64 of unknown phyletic selectivity. Of the insecticidal spider toxins, the molecular target has only been identified for 85 (63%). To date, the most common identified targets of insecticidal spider-venom toxins are Na_v_ channels (n = 33), Ca_v_ channels (n = 33), the lipid bilayer (n = 11), calcium-activated potassium (K_Ca_) channels (n = 7), presynaptic nerve terminals (n = 2) and N-methyl-D-aspartate (NMDA) receptors (n = 1) [24]. These toxins were isolated from the venom of 20 of the 110 extant spider families, including representatives from the two major infraorders Araneomorphae (“modern” spiders) and Mygalomorphae (“primitive” spiders). Araneomorphs represent >90% of all known spider species. In recent years, it has become clear that spider venoms are considerably more complex than previously realized, with some venoms containing more than 1,000 distinct peptides [24]. Assuming 100,000 species and 200 peptides per venom, then spider venoms may contain upwards of 10 million bioactive peptides. Less than 0.01% of this proteomic diversity has been explored to date [26].

Spiders utilize their venoms to paralyze and/or kill prey or predators as rapidly as possible. Therefore their venoms are particularly rich in neurotoxins targeting the insect nervous system that rapidly modify ion conductance (ion channel toxins) and, to a lesser extent, affect neurotransmitter exocytosis (presynaptic toxins). Many of these spider peptide toxins are selectively insecticidal. In particular, insect-selective toxins have been patented for their possible use as bioinsecticidal agents for the control of phytophagous pests or insect vectors. Spider-venom peptides are a rich source of potential bioinsecticides that can combine the desirable attributes of high potency, novel target activity, structural stability and phyletic selectivity [27]. Moreover, pharmacological characterization of spider toxins is revealing novel target sites not previously exploited by conventional agrochemicals, thereby validating new insecticide targets for future screening programs. These peptides can be delivered to insect pests via many different routes, including incorporation of transgenes encoding the peptides into entomopathogens or crop plants [28]. To be commercially successful, venom peptides must have broad pest-species specificity, show low toxicity toward non-target organism, not induce resistance in the pest species, be inexpensive to produce in large quantities, and be convenient to apply [26].

## 4. Development of Recombinant Venom Peptide for Pest Control

### 4.1. Selection of Spider Venom Peptide for Potential Pest Control Application

The selective spider venom peptide, lycotoxin-1 (also known as lycotoxin-I), from wolf spider (*Lycosa carolinensis*) venom demonstrates both antimicrobial and insect neuroactive properties [8]. From its amphipathic nature and physiological actions lycotoxin-1 appears to function as a pore former to increase membrane permeability (typical of CPP-like MAMPs), dissipate voltage gradients, and induce lysis of insect cells [8]. These properties suggest the potential use of lycotoxin-1 as a bioinsecticide. Although peptides with more potent insecticidal activity have been reported recently in the literature [26], lycotoxin-1 was selected because its sequence was known and its short length suggested that correctly folded variants of the lycotoxin‑1 peptide with optimized insecticidal activity for oral or topical application could potentially be produced by mutagenesis of the gene for the naturally occurring peptide. Antimicrobial peptides from a related species of *Lycosa* indicate some variation in peptide sequence is possible without losing biological activity [29]. A search of the ArachnoServer database that is now available [24,25].would undoubtedly identify candidates that are more promising.

The mechanism of interaction of MAMPs with cell membranes does not involve specific protein binding sites, which reduces the likelihood of inducing drug resistance. Specificity is much less of an issue when the toxin is expressed in the plant because most beneficial insects typically do not feed on the host plant. Use of tissue-specific promoters such as the chlorophyll a/b binding protein promoter [30] to prevent expression in pollen also helps to limit the toxin to tissues that are not consumed by beneficial insects. For oral or topical delivery, methodology can be designed to selectively target the pest insects, for example, by using attractants (such as pheromones) to draw them to the toxin or by incorporating the toxin into starch or flour that is applied as a bait with a feeding stimulant for the pest insect. Molecular evolution can also be used to develop more effective variants.

### 4.2. Optimization of Lycotoxin-1 Sequence and Lethality of Mutants to Armyworms

Initial assays with lycotoxin 1 indicated it was inactive when fed to fall armyworms, a major insect pest of corn and other crops in the United States. Presumably this inactivity was the result of its inability to penetrate to the target site because of the oral delivery route. Normally spiders inject the peptides into their prey with their fangs, thereby bypassing the physical barrier of the insect cuticle. To investigate whether changes in the primary structure of lycotoxin-1 peptide might improve its efficacy as an oral or topical bioinsecticide, a gene library expressing recombinant mutant lycotoxin-1 peptides was produced using an amino acid scanning mutagenesis strategy [31] and screened in high throughput for ability to kill fall armyworms. Yeast cultures expressing the recombinant peptide toxins were fed to armyworm larvae to identify the mutant toxins with the greatest lethality. One culture that was highly lethal to the armyworms contained a yeast strain expressing a peptide with the following sequence: H_2_NHHHHHHDDDKIWLTALKFLGKHAAKHLAKQQLSPWCOOH. The 6xHis site was used for purification and the EntK site (DDDK) was designed to be cleaved by trypsin in the insect midgut to release the active 25-amino acid lycotoxin-1 peptide variant in the cuticle-lined gut region and allows for penetration into the insect. This variant differed from wild-type lycotoxin-1 in the two amino acids at the carboxy terminus, with proline and tryptophan residues replacing the lysine and leucine residues at positions 24 and 25, respectively, in the wild-type lycotoxin-1.

The lycotoxin-1 variant that was highly lethal to the fall armyworms in this initial evaluation was selected for further optimization by amino acid scanning mutagenesis [11]. The lycotoxin variants produced by further optimization were again screened for lethality to armyworms. Sequence analysis of the three most lethal toxins, A6, B9, and C6, indicated mutations at position 8 in A6 from leucine to histidine, at position 10 in B9 from glycine to glutamine, and at position 9 in C6 from leucine to serine. Additionally, in clone B9 there are PCR mutations in position 20 and 21 putting histidine and serine, respectively in place of two glutamine residues. All three of the most lethal clones also have mutations in positions 24 and 25 from lysine and leucine to proline and tryptophan, respectively.

**Figure 1:**
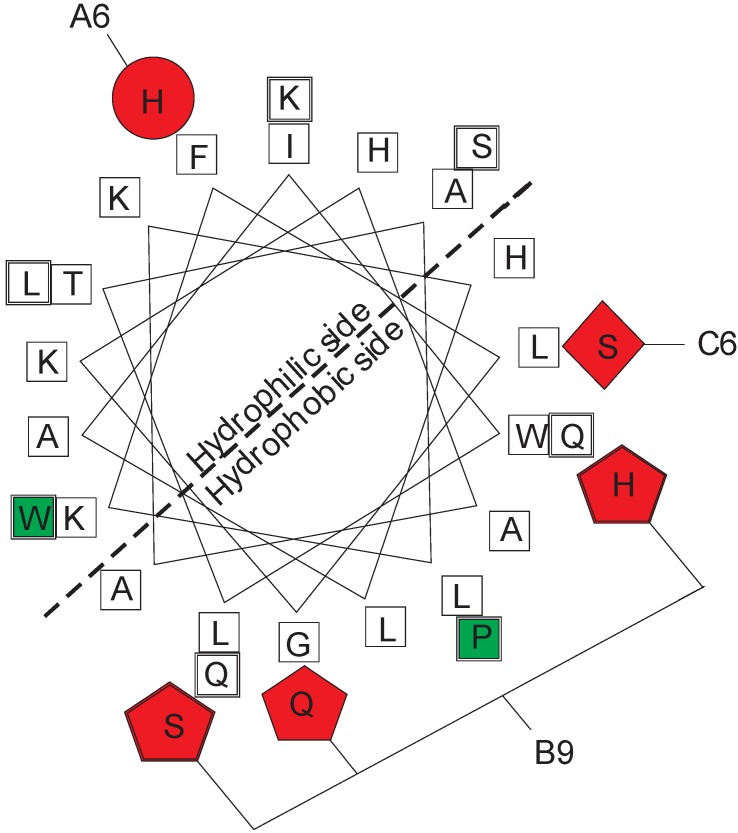
Helical-wheel projection of the amphipathic lycotoxin-1 peptide and the position of the lycotoxin-1 mutations in the hydrophobic and hydrophilic portions of the three-dimensional structure.

A helical-wheel projection of the amphipathic peptide illustrates the lycotoxin-1 mutations relative to the hydrophobic and hydrophilic sections of the three-dimensional structure (Figure 1 source from [11]). All variants have mutations Lys24Pro and Leu25Trp indicated by the green squares (wild-type amino acid residues are not shown at those positions in wheel). Red symbols indicate additional mutations in A6 (Phe8His circle), B9 (Gly10Gln, Gln20His, Gln21Ser pentagons), and C6 (Leu9Ser diamond). None have mutations in the lysine residues in the hydrophilic section.

The mutations in the lycotoxin-1 variants that showed the greatest lethality appear to modify the amphipathic properties of the peptide structure by altering charge, reducing or increasing steric effects, and/or changing polarity. Specifically, the substitutions of proline and tryptophan replacing lysine and leucine at positions 24 and 25, respectively, have possible steric and charge effects. The change at position 24 increases the hydrophobic nature of the hydrophobic side of the helix (Figure 1) and ensures two hydrophobic residues at the C-terminal, which have been found to be important for activity [32], although the exact functional significance is unknown. Tryptophan residues in conjunction with lysines have also been shown to contribute to favorable interaction with negatively charged cell membranes [32]. The substitution of a polar Ser residue for a neutral Leu residue at position 9 may increase the interaction with the interior of the cell membrane. The positively charged lysine core, with Lys residues at positions 7, 11, 15, and 19, is unchanged in the individual variants that showed the greatest lethality, and this region is increased further in positive charge in optimized mutant A6, which has a phenylalanine to histidine mutation at position 8. This positively charged region is crucial for binding to negatively charged insect cell membranes [32].

### 4.3. Expression of Spider Toxins in Transgenic Plants

A small number of transgenic plants expressing spider toxins have been created in the last few years. The w-ACTX-Hv1a toxin gene from the Australian funnel web spider (*Hadronyche versuta*), which codes for a 37-amino-acid calcium channel antagonist, was synthetically produced with codon optimization for plants and constitutively expressed in all tobacco tissues [33]. These transgenic tobacco plants caused 100% mortality of two kinds of caterpillars, cotton bollworm, *Helicoverpa armigera*, and cotton leafworm, *Spodoptera littoralis*, within two days. The same toxin from *Hadronyche versuta* was also effective against cotton bollworm, *Helicoverpa armigera,* caterpillars when expressed only in the phloem of transgenic tobacco [34]. A spider toxin from *Macrothele gigas*, predicted to be a 38-amino-acid mature peptide, with insecticidal properties but an unknown mode of action, was expressed in transgenic tobacco that conferred resistance to fall armyworm caterpillars [35]. Two of these published studies reported that expression of the spider toxin caused no morphological abnormalities in the transgenic plants [33,35], indicating the toxins do not have plant targets. These published studies demonstrate that transgenic plant expression of spider toxin peptides for insect control is feasible. In experiments in our laboratories, putative transgenic tobacco plants expressing a lycotoxin-1 peptide variant (sequence given in Section 4.2) exhibited increased leaf resistance to feeding by corn earworm and cigarette beetle larvae compared to wild type plants. Experiments are in progress to determine the concentration of lycotoxin-1 variant in the most biologically active plants.

## 5. Conclusions

Pest control is just one of the many potential uses of CPPs and CPP-like molecules. Recombinant technology and high-throughput platforms enable the rapid screening and selection of the most effective agent against a target pest. The possibility of encoding transgenes in a plant that serve a protective function against plant pests is a promising start in developing viable alternative biostrategies for plant pest control. For commercial success, it remains to be demonstrated that the potential agent is safe for non-target organisms, that it does not induce resistance, and that it can be produced inexpensively and in adequate quantities. However, with millions of possible bioactive peptides in the venom of spiders, and the application of molecular techniques to optimize efficacy and selectivity, an almost inexhaustible source of candidates is available.
